# The relationships of OSBPL3 expression with KI-67 expression and KRAS mutations in CRC: implications for diagnosis and prognosis

**DOI:** 10.1186/s12920-022-01402-w

**Published:** 2022-12-14

**Authors:** Min Zhang, Lei Meng, Zhaoxuan Zhang, Jing Wu, Xi Chen, Yuejing Wang, Jie He

**Affiliations:** 1 Department of Pathology, The First Affiliated Hospital of USTC, Division of Life Sciences and Medicine, University of Science and Technology of China, Hefei, 230001 Anhui China; 2 Department of Gastrointestinal Surgery, The First Affiliated Hospital of USTC, Division of Life Sciences and Medicine, University of Science and Technology of China, Hefei, 230001 Anhui China; 3 Anhui Medical University, Hefei, Anhui China

**Keywords:** Colorectal cancer, CRC, OSBPL3, Ki-67, KRAS, Prognosis

## Abstract

**Background:**

OSBPL3 is overexpressed in a variety of malignancies and is closely associated with tumor growth and metastasis. However, its expression and function in colorectal cancer (CRC) are unclear. We aimed to investigate its prognostic and therapeutic value in this disease by detecting its expression in CRC and its correlation with the clinicopathological characteristics and prognosis of patients.

**Methods:**

A total of 92 CRC samples were included in this study. According to the 2020 WHO diagnostic criteria, the criteria of the American Joint Committee on Cancer (AJCC) 8th edition staging system were used. OSBPL3 and Ki-67 expression in these samples was detected by immunohistochemistry. OSBPL3 mRNA expression was detected by qRT-PCR. KRAS/NRAS mutations were detected by an amplification refractory mutation system (ARMS). Data analysis was performed using the statistical analysis software Prism 8.

**Results:**

OSBPL3 was found to be significantly overexpressed in CRC tumor tissues and was associated with worse progression-free survival and overall survival in patients. Additionally, OSBPL3 expression was negatively correlated with the degree of tumor differentiation. KRAS mutations were detected in approximately 32.6% of patients and were significantly associated with high OSBPL3 expression. In addition, OSBPL3 and Ki-67 expression was significantly correlated.

**Conclusions:**

OSBPL3 is highly expressed in CRC samples and predicts a worse prognosis. OSBPL3 may become a new potential therapeutic target for CRC.

**Supplementary Information:**

The online version contains supplementary material available at 10.1186/s12920-022-01402-w.

## Background

In 2020, there were more than 1.9 million new cases of CRC and approximately 935,000 related deaths worldwide, accounting for one-tenth of cancer cases and related deaths, and according to the International Agency for Research on Cancer, CRC ranks third in incidence and second in mortality [1]. It ranks second in cancer incidence in men and third in women. Data from the National Cancer Center of China in 2019 showed that there were 388,000 new cases of CRC and 187,000 deaths in China in 2015, accounting for 9.87% of diagnoses and 8.01% of related deaths. Effective screening measures, early intervention and better treatment measures can reduce the mortality rate of patients [2]. The main treatments currently available are surgery, chemotherapy, targeted therapy and immunotherapy [3]. The 5-year relative survival rate for CRC patients is 65%, and that of rectal cancer (67%) is slightly higher than that of colon cancer (64%) [4]. The 5-year relative survival rates for stage I and II patients are 91% and 82%, respectively, while the 5-year survival rate for stage IV patients is only 12%. Approximately 90% of CRCs are adenocarcinomas in terms of pathological type, while rare types include mucinous adenocarcinoma, signet ring cell carcinoma and myeloid carcinoma. Immunohistochemical detection of the Ki-67 index is an independent factor affecting the prognosis of CRC, and high Ki-67 staining is strongly associated with a poor prognosis in CRC, is positively correlated with CRC invasion depth, lymph node metastasis, and tumor differentiation, and is an independent predictor of prognosis that can be used to stratify patients [5].

Studies on the driver genes and prognostic and predictive molecular mechanisms of CRC have shown that RAS genes are representative of established biomarkers for efficacy prediction and prognostic risk assessment. RAS belongs to a class of GTPase proteins that regulate signaling pathways that control processes such as cell proliferation, cell differentiation, cell adhesion, apoptosis and cell migration. The invasive and metastatic potential of cells is increased when RAS is mutated. The main members of the RAS family are KRAS and NRAS [6]. The KRAS gene is one of the most common oncogenes in solid tumors, and it is mutated in 81.35% of pancreatic cancers and 48.33% of CRCs [7]. KRAS mutation is the main driver of colon cancer [6]. Mutations in the KRAS oncoprotein impair its intrinsic GTP hydrolase activity, locking it in its GTP-bound active state, and thus abnormally stimulate the RAS-RAF-MEK-ERK (MAPK) signaling pathway [8]. A mutated RAS gene, which is not regulated by EGFR expression signals, automatically activates its downstream signaling pathways, enabling tumor growth and proliferation [9].

Oxysterol-binding protein (OSBP) and its related proteins (oxysterol-binding protein-related protein (ORP) or OSBP-like proteins (OSBPLs) constitute a conserved family of lipid transfer proteins (LTPs) [10]. One of its members, OSBPL3 (ORP3), is expressed mainly in the brain, kidney, spleen, lymphoid tissue and leukocytes [11, 12] and is significantly more highly expressed in 21 malignancies, including CRC, than in normal controls [13, 14]. Because of its key role in controlling cell adhesion and migration, it is expected to be a drug target for tumor therapy [10]. However, its tumor suppressive function has also been reported [15].

Overexpression of OSBPL3 promotes the proliferation, invasion and metastasis of CRC in vitro and in vivo. The expression level of OSBPL3 in CRC was found to be positively correlated with poor differentiation, tumor-node-metastasis (TNM) stage and Dukes stage [14]. It has also been suggested that the OSBPL3 mRNA level may be a prognostic marker for better stratification of CRC patients [16]. Bioinformatic analysis revealed that OSBPL3 promotes CRC progression by activating the RAS signaling pathway [14]. Hyperphosphorylated OSBPL3 interacts with the endoplasmic reticulum membrane protein VAPA to generate OSBPL3-VAPA complexes to stimulate R-Ras signaling [17]. Overexpression of OSBPL3 leads to the formation of polarized cell surface protrusions, impaired cell spreading, and reduced integrin activity, and OSBPL3 acts upstream of the RAS and may mediate cell matrix adhesion by regulating integrin activity, thereby altering RAS activity [17, 18]. In this study, we detected the mRNA and protein expression of OSBPL3 and Ki-67 in 92 CRC tissues and cancer-adjacent normal tissues by immunohistochemistry and qPCR and detected RAS gene mutation by the amplification refractory mutation system (ARMS) method to initially explore the role of OSBPL3 and the RAS signaling pathway in CRC progression and provide a theoretical basis for therapeutic target screening.

## Methods

### Sample information

Paraffin samples, including CRC tissues and adjacent normal tissues, were selected from 92 patients who underwent radical abdominal surgery in Anhui Cancer Hospital between April 2014 and December 2016, which were histopathologically confirmed to have CRC and had complete molecular pathology data. Among the patients, 49 had colon cancer and 43 had rectal cancer. Their ages ranged from 20 to 79 years, with a median age of 60 years, and detailed clinical information is shown in Table 1. The histological grades of the tumors were classified according to the percentage of adenoid structure formation and differentiation status. Lymph node metastasis was classified as negative (no regional lymph node metastasis) or positive (metastasis to regional lymph nodes). Tumor stage was classified according to the TNM staging system of the American Joint Committee on Cancer (AJCC) and was divided into stage I-II and stage III-IV. The study was approved by our ethics committee, and for survival analysis and follow-up, the date of surgical resection was used as the beginning of the follow-up date. The absence of informed consent, withdrawal of consent by the patient, and incompletion of histopathological or molecular pathological information all could be the exclusion criteria. Patients who died from diseases other than CRC or died from unexpected events were excluded from the survival analysis. The follow-up period was 4 to 6 years.

**Table 1:** Clinical characteristics of 92 CRC cases

Characteristics	Characteristics Number of patients (%)
Total	92
Age	
Median (years)	60, 49–64
Sex	
Male	62 (67.39)
Female	30 (32.61)
Status	
Alive	47 (51.09)
Dead	40 (43.48)
Lost contact	5 (5.43)
Location	
Right	19 (20.65)
Left	31 (33.70)
Rectum	42 (45.65)
TNM stage	
Stage I	3 (3.26)
Stage II	35 (38.04)
Stage III	49 (53.26)
Stage IV	5 (5.43)
Tumor size	
T1	0(0)
T2	4 (4.35)
T3	22 (23.91)
T4	66 (71.74)
Lymph-node metastasis	
N0	40 (43.48)
N1-2	52 (56.52)
Distant metastasis	
M0	88 (95.65)
M1	4 (4.35)
Differentiation	
Well	64(69.57)
Poor	28(30.43)
With intestinal polyps	
Yes	12 (13.04)
No	80 (86.96)

Values provide the median with IQR

### OSBPL3 mRNA levels by qRT-PCR

Total RNA extraction: Depending on the size of the paraffin-embedded tissues, 3–6 white slices of 5–10 µm thickness were cut and dewaxed, tumor cells were enriched (control tissues did not need this step) and digested, and total RNA from paraffin-embedded tissues was extracted using the OMEGA RNA Isolation Kit (Cat. No. R6954-02, OMEGA, USA). The concentration and purity of the extracted RNA were determined using a BioDrop ultramicro nucleic acid protein analyzer, requiring an RNA A260/A280 between 1.8 and 2.0, and stored at − 20 °C. Then, the High Capacity cDNA Reverse Transcription Kit (Lot No. 00307397, Part No. 4368813, Applied Biosystems by Thermo Fisher Scientific, USA) was used for reverse transcription. qRT-PCR was performed on an Applied Biosystem 7500 PCR instrument with Power SYBR Green PCR Master Mix (Lot No. 1711564, Part No. 4367659, Applied Biosystems by Thermo Fisher Scientific, USA) based on the manufacturer’s scheme. All samples were processed under the same experimental conditions. Primers were synthesized by Shanghai Shiny Crystal Molecular Biotechnology Co. The primer sequence are listed as follows: β-actin-F: AGCCATGTACGTTGCTATCCA; β-actin-R: GTCACCGGAGTCCATCACGAT; OSBPL3-F: GCCTGTCCTTGATAGTGGTCG; OSBPL3-R: CGTGTTCAGGGGCTCGTTC. The cycling conditions were as follows: preincubation at 95 °C for 5 min, denaturation at 95 °C, annealing at 62 °C, extension at 72 °C for 10 s each, 40 cycles; cooling at 40 °C for 30 s. Each reaction well was repeated 3 times. β-Actin was used as an internal reference to calculate the relative 2-∆∆CT values.

### Immunochemistry and evaluation

The expression of the OSBPL3 gene at the protein level was examined by immunohistochemistry (IHC) in patient's paraffin samples. A polyclonal rabbit anti-OSBPL3 antibody (1:100; NBP1-82968; NOVUS, USA) and a monoclonal rabbit anti-Ki-67 antibody (ready-to-use; 790-4286, clone number 30–9; Roche, USA) were used to detect the corresponding proteins. Immunostaining was performed on a Roche Benchmark XT automated staining system (Roche/Ventana) according to the instructions. Procedure: Three-micrometer sections were dewaxed in EZprep concentration buffer at 75 °C for 4 min. Epitope repair was performed in cell conditioning solution at 100 °C for 64/76 min. Anti-OSBPL3 and anti-Ki-67 were both incubated at 37 °C for 32 min. Then, goat anti-mouse/anti-rabbit IgG/IgM secondary antibody coupled with horseradish peroxidase was added for 8 min followed by DAB visualization and finally hematoxylin staining. The independent individuals observed the samples separately, without prior knowledge of either patient's clinical information or outcomes. Differences between the two assessors were resolved by reassessment and discussions until agreement were reached. During the process, the microscope model was KF-PRO-120 digital section scanner; the objective lenses model was OLYPUS flat-field compound achromatic objective and 0.75 bore diameter; the camera was 3CCD linear camera; and the KScanner acquisition software was used. OSBPL3 analysis: Color development was localized to the nucleus and the plasma membrane. A brownish-yellow color of the nucleus or pulp was considered positive. The immune response score (IRS) was calculated based on the staining intensity (SI) multiplied by the percentage of positively stained cells (PP). The specific scoring criteria were as follows. SI (range 0–3): 0 was negative, 1 was weak, 2 was moderate, and 3 was strong staining; PP (range 0–4): 0 indicated 0%, 1 indicated 1% to 25%, 2 indicated 26% to 50%, 3 indicated 51% to 75%, and 4 indicated 76% to 100% positively stained cells. IRS scores ranged from 0 to 12. OSBPL3 was considered highly expressed if the IRS was 8–12 and expression at low levels if the IRS was 0–7. Ki-67 analysis: Color development was localized to the nucleus. A brownish-yellow nucleus was considered positive. The proportion of Ki-67-positive cells to total tumor cells was assessed in 10 representative high magnification fields of tumor cells (screening 100 cells in the upper, lower, left, right, and central fields), and in this study, a set cutoff value 60% was used: < 60% was considered low expression, and ≥ 60% was considered high expression.

### RAS gene mutation by ARMS

Pretreatment of paraffin-embedded tissues was performed as previously described. Genomic DNA was extracted from paraffin-embedded tissues using the QIAamp DNA FFPE Tissue Kit (Cat No. 56404, QIAGEN, Germany). In addition, a BioDrop ultramicro nucleic acid protein analyzer was used to determine DNA purity with an A260/A280 ratio between 1.8 and 2.0. The samples were diluted to the appropriate concentration for subsequent testing. The Human KRAS Gene Mutation Detection Kit (PCR-Fluorescent Probe Method) and the Human NRAS Gene Mutation Detection Kit (PCR-Fluorescent Probe Method) (Wuhan Youzhiyou Medical Technology, China) were used to detect KRAS/NRAS mutations in all samples on an Applied Biosystems 7500 PCR instrument. Negative, weakly positive and blank control samples were set up.

### Statistical analysis

Data were analyzed using Prism 8. *P* values for assessing the significance of differences between Kaplan–Meier survival curves were estimated using the log-rank test. *P* < 0.05 was considered to indicate statistical significance. Correlation analysis of clinicopathological parameters with OSBPL3 expression was performed using Fisher's exact test, and *P* < 0.05 was considered to indicate statistical significance. Histograms and box plots were calculated for statistical analysis (two tailed Student's t-test for both groups).

### Comparison of OSBPL3 status by public database

Based on the TCGA (Cancer Genome Atlas) and GEO (Gene Expression Comprehensive Database) datasets, the expression differences of OSBPL3 in 33 tumors were compared. The tumor tissues and the corresponding normal tissues were compared by the GTEx (Genotype-Tissue Expression) database with *P* value cutoff = 0.01, log2FC (fold change) cutoff = 1 and "Match TCGA normal and GTEx data" settings, the UALCAN portal (http://ualcan.path.uab.edu/analysis-prot.html) was used to analyze canceromics data resources and to compare the differences in mRNA and protein expression between CRC tissues and normal tissues. The survival data site (http://www.oncolnc.org/), which contains information on 21 tumors, was used to query and to analyze the survival differences between the high and low OSBPL3 expression groups in CRC.

## Results

### OSBPL3 overexpression promotes CRC progression

**Fig. 1:**
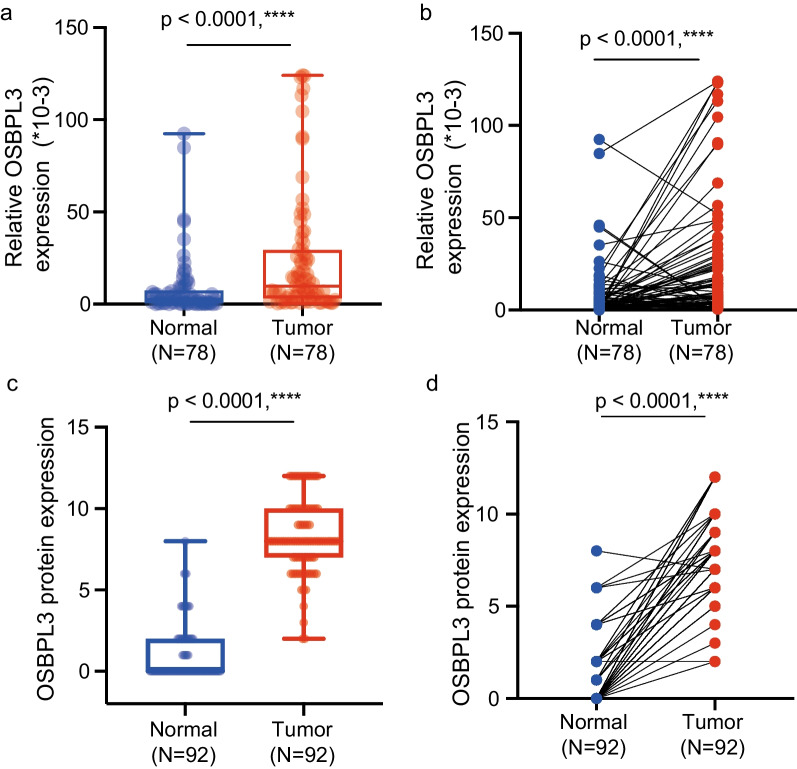
OSBPL3 mRNA and protein expression in pairs of paraffin CRC tissues and paired normal tissues by real-time PCR and immunohistochemistry. **a**, **b** Showed that OSBPL3 mRNA expression levels were significantly higher in colorectal cancer tissues compared with paired normal tissues from the same patients. **c**, **d** Showed that OSBPL3 protein expression levels were significantly higher in colorectal cancer tissues compared with paired normal tissues from the same patients (Magnification × 400)

**Fig. 2:**
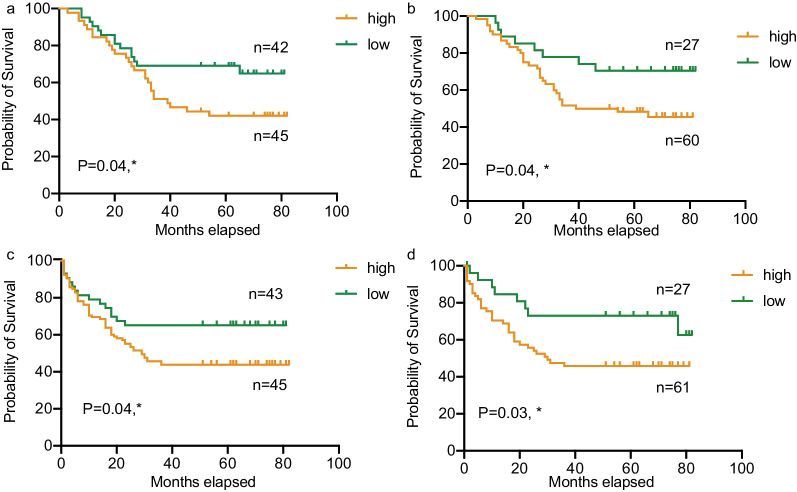
Kaplan–Meier curves were used of progression free survival and overall survival in high and low risk groups for CRC patients. The cutoff values for the high and low risk groups were based on the median of the risk score. **a** The correlation between OSBPL3 mRNA and progression free survival in CRC patients. **b** The correlation between OSBPL3 protein levels and progression free survival in CRC patients. **c** The correlation between OSBPL3 mRNA and the overall survival time of CRC patients. **d** The correlation between OSBPL3 protein levels and the overall survival time of CRC patients. Prognosis was significantly worse for patients with high expression of either OSBPL3 mRNA or protein, compared with patients with low expression. Green: high OSBPL3 expression groups; Orange: low OSBPL3 expression groups

**Fig. 3:**
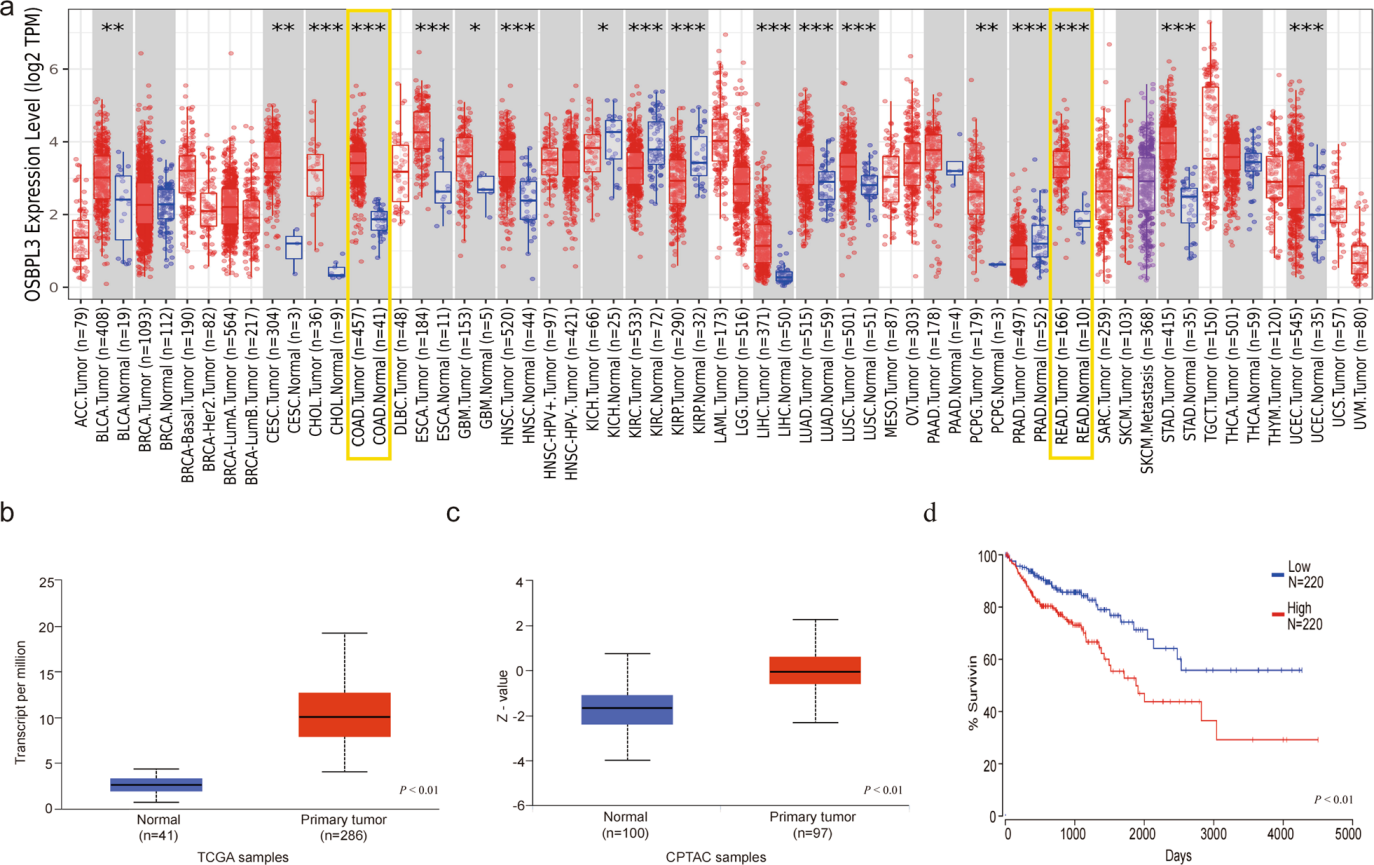
Bioinformatics data of OSBPL3 overexpression promoting CRC progression. **a** Pan-cancer analysis of mRNA expression of OSBPL3 in TCGA database, Colon and rectal cancers tissues and normal tissues are highlighted in yellow boxes respectively, the results are significantly different (***P < 0.001). **b** Differences in mRNA expression of OSBPL3 in normal intestinal tissues and tumor tissues in TCGA database (**P < 0.01). **c** Differences in protein expression of OSBPL3 in normal tissues and tumor tissues in GPTAC database (**P < 0.01). **d** Differences in overall survival time of CRC patients between high and low OSBPL3 expression (**P < 0.01)

qRT-PCR of paraffin specimens from 78 CRC patient tissues and adjacent normal tissues showed that OSBPL3 mRNA levels were significantly higher in cancer tissues than in normal tissues (*P* < 0.0001) (Fig. 1a, b). Immunohistochemical assays of 92 CRC samples also showed that OSBPL3 were highly expressed in cancer tissues (*P* < 0.0001) (Fig. 1c, d). Progression-free survival (PFS) and overall survival (OS) were significantly worse in CRC patients with high OSBPL3 expression than in those with low OSBPL3 mRNA or protein expression (*P* < 0.05) (Fig. 2). The results suggest that OSBPL3 promotes the malignant progression of CRC and that high OSBPL3 expression is associated with a poor prognosis. This is consistent with the results of data already available in public databases (Fig. 3).

### High OSBPL3 protein expression correlates with poor CRC differentiation

**Fig. 4:**
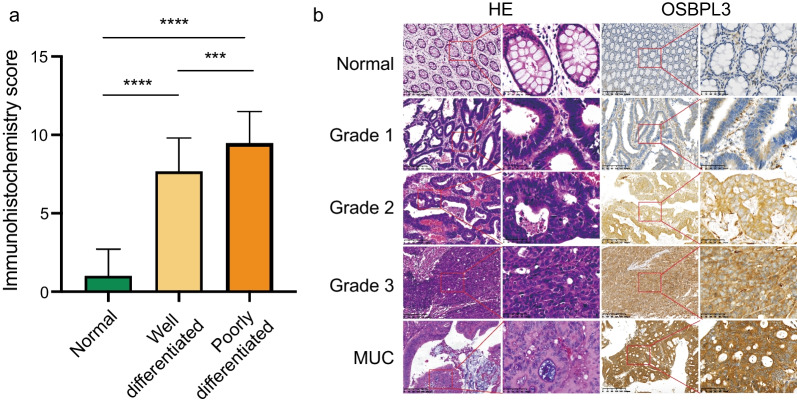
Relationship between OSBPL3 protein expressions and differentiations. **a** There were significant difference in the expression of OSBPL3 between tumor and normal tissues (****P < 0.0001); the expression of OSBPL3 in tumor tissues was negatively correlated with the degree of tumor differentiation from poor to well. With the decrease of differentiation degree, the scores of OSBPL3 protein expressions tend to be higher, and there were a statistical significance in differentiation (***P < 0.001). **b** Representative results of OSBPL3 by IHC. It was weakly expressed in cytoplasm and cell nucleus of normal intestinal epithelium and well differentiated tumor tissues; it was highly expressed in cytoplasm of poorly differentiated tumor tissues (Magnification × 200, × 400)

To investigate the relationship between differential OSBPL3 expression and clinicopathological parameters of CRC patients, statistical analysis of mRNA expression was first performed and found that mRNA expression levels correlated with the degree of tumor differentiation (Table 2, *P* < 0.05). There were no significant relationships between mRNA expression levels and clinicopathological parameters such as tumor size, lymph node metastasis, sex, age, presence of concomitant polyps, left and right halves, etc. Further statistical analysis of the OSBPL3 immunohistochemical results was performed. The results showed that the expression of OSBPL3 in tumor tissues was negatively correlated with the degree of tumor differentiation (Fig. 4a). Significant differences in OSBPL3 immunohistochemical scores were found in normal paracancerous tissues, highly differentiated tumor tissues, and poorly differentiated tumor tissues (*P* < 0.001), with increasing intensity of expression in ascending order. It was weakly expressed in the cytoplasm and nucleus of normal intestinal epithelium and highly differentiated tumor tissues and strongly expressed in the cytoplasm and nucleus of poorly differentiated tumor tissues (Fig. 4b). The results suggest that OSBPL3 can be used as a specific molecular indicator of CRC differentiation and is expected to be a new molecular target.

**Table 2:** Correlation of OSBPL3 with clinicopathological parameters of CRC patients

Variables	OSBPL3 mRNA levels		*P* value
	Low	High	
Age			
≤ 60	27	19	0.053
> 60	23	23	
Sex			
Male	31	31	0.069
Female	15	5	
Location			
Colon	22	20	0.83
Rectum	24	26	
TNM stage			
I–II	23	17	0.29
III–IV	23	29	
Tumor size			
T1–T3	13	13	> 0.99
T4	33	33	
Lymph-node metastasis			
N0	22	18	0.52
N1-2	24	28	
Distant metastasis			
M0	44	44	> 0.99
M1	2	2	
Differentiation			
Well	38	26	0.02*
Poor	8	20	
Polyps			
Yes	5	7	0.75
No	41	39	
KRAS mutation			
Wild type	37	24	0.007**
Mutant	9	21	

*P* was calculated using Fisher’exact test. *P < 0.05, **P < 0.01

### The protein expression levels of OSBPL3 and Ki-67 in CRC tissues were significantly correlated

**Fig. 5:**
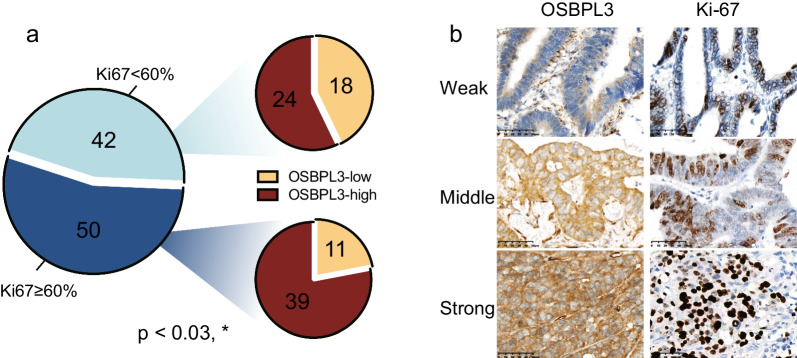
Expression of OSBPL3 and Ki-67 in CRC samples detected by immunohistochemistry. **a** The pie chart shows the number of co-expression OSBPL3 and ki-67 in CRC samples. **b** Representative image of OSBPL3 and ki-67 score from weak to strong on normal intestinal epithelium and tumor tissues. Staining location: OSBPL3 staining is located in the nucleus and cytoplasm, and Ki-67 staining is located in the nucleus. The expression of Ki-67 was also weak in tissues with weak OSBPL3 expression; Ki-67 expression was also strong in tissues with strong expression of OSBPL3 (Magnification × 400)

To explore the relationship between differential OSBPL3 expression levels and the tumor Ki-67 index in CRC patients, further statistical analysis of the immunochemical results of both was performed. Notably, we found a strong concordance between OSBPL3 overexpression and high Ki-67 expression in tumor cells within most tissue sections that showed immunoreactivity for both OSBPL3 and Ki-67, and OSBPL3 overexpression was strongly associated with high Ki-67 expression (Fig. 5, *P* < 0.05). This further indicates that OSBPL3 could be a new molecular target.

### Association between the KRAS mutation and OSBPL3 expression

KRAS mutations were detected in 30 of 92 CRC cases (32.6%), and the main mutation sites were G12D (13/30, 43.3%), G13D (6/30, 20%), and G12 V (6/30, 20%) in codons 12 and 13 of exon 12, while other mutations, such as G12C (2/30, 6.7%), G12A (2/30, 6.7%), and G12S (1/30, 3.3%), were rare. The expression level of OSBPL3 was significantly elevated in KRAS mutant tumors (Table 2, *P* < 0.01), but there was no significant correlation between OSBPL3 expression and each subtype of KRAS mutation. The results showed that CRC samples with high OSBPL3 expression had more KRAS mutations, again confirming the potential of OSBPL3 as a new molecular target.

## Discussion

The early diagnosis of CRC and timely surgery support a good prognosis, but approximately 25% of CRC cases are already advanced at the time of diagnosis [19]. Some still have surgical opportunities after neoadjuvant radiotherapy to reduce the clinical stage, and supplementation with targeted therapy or immunotherapy significantly prolongs survival [20]. Therefore, CRC survival can be improved by means of precision therapy regardless of disease stage, and CRC is curable if early diagnosis can be achieved. Thus, early diagnostic molecular marker screening is particularly important, and molecular target selection provides a reliable basis for targeted therapy. Thus, CRC molecular markers have become a research hotspot in recent years.

The results of this study showed that OSBPL3 expression was significantly higher in CRC tissues than in paraneoplastic tissues and correlated with the degree of CRC differentiation; the lower the CRC differentiation, the higher the OSBPL3 mRNA and protein expression, which is consistent with the results of existing studies and biochemical predictions [14]. It has also been shown that the degree of differentiation does not directly correlate with OSBPL3 expression, but rather the prognosis differs between groups with high and low expression in tumors with different degrees of differentiation [16]. In the weighted gene correlation network analysis (WGCNA) study, OSBPL3 was identified as a pivotal gene in CRC, with upregulated expression in cancer tissues, and its high expression correlated with a poor prognosis in CRC [21]. The results of the present study support the association of OSBPL3 with CRC development and progression. OSBP and OSBP-associated proteins (ORPs) constitute a large family of genes with sterol/lipid transport and regulatory activities involved in the control of lipid metabolism, regulation of vesicular transport and cell signaling events [22, 23]. ORP4, ORP5 and many other members of the ORP family have also been associated with tumors. ORP4 promotes the survival of rapidly proliferating cells [24] and is considered a potential marker of solid tumor dissemination and a poor prognosis [25]. Highly spliced variants leading to small changes in mRNA structure have been identified in several ORPs, including ORP1, ORP3 and ORP6 [26]. Differential mRNA splicing may result in functionally different forms of the OSBPL3 protein [11]. Jiao et al. confirmed that OSBPL3 promotes the proliferation, migration, and motility of CRC cells by ex vivo experiments [14].

In the present study, we found that those who overexpressed OSBPL3 in CRC had correspondingly high expression of Ki-67, and there was a positive correlation between them. Ki-67 is a nuclear DNA-binding protein expressed in all vertebrates and is a proliferation marker widely used for tumor grading [27]. Ki-67 is present in the G1, S, and G2 phases of the cell cycle and is commonly used as a marker of cell proliferation [28]. Immunohistochemical detection of the Ki-67 index in tumors can objectively reflect the proliferation of tumors and is well established for clinical application. A high Ki-67 index usually indicates active proliferation and poor prognosis [29]. One study confirmed that the positive expression of Ki-67 in colorectal cancer increased with a decrease in differentiation [30]. High Ki-67 expression indicates a lower survival rate and is a predictor of CRC progression [31]. In this study, we concluded that OSBPL3 and Ki-67 expression were correlated, which indicates that OSBPL3 may have a pro-proliferative effect on CRC, which was positively correlated with the degree of differentiation. Therefore, it was further hypothesized that OSBPL3 and Ki-67 have the same pro-proliferative function and that high OSBPL3 expression is associated with a poor prognosis and could be used as a marker of CRC cell proliferation.

This study also found that KRAS mutations were more common in cases with high OSBPL3 expression, and the two were closely related. However, there was no clear relationship between OSBPL3 expression levels and KRAS mutation subtypes. Considering the effect of the small sample size, the variation in mutant subtypes needs further validation. RAS is an oncogene that plays a crucial role in cell proliferation, differentiation, growth and development [8]. Cyclin D1, the downstream target gene of its downstream signaling pathway Ras/Raf pathway, is a key factor in controlling cell proliferation from G1 to S phase and ultimately promoting cell proliferation [32]. It has been suggested that OSBPL3 is an R-Ras interacting oxysterol-binding protein homolog that regulates cell adhesion and plays a role in promoting tumor cell proliferation, migration and invasion, and evidence was obtained that the OSBPL3-VAPA complex stimulates R-Ras signaling [17]. It can regulate cytoskeleton reconstruction, alter the shape of CRC cells and the number of laminar pseudopods, and promote the motility and migration of CRC cells [14]. KRAS mutations are common driver mutations in CRC and are found at different frequencies in all consensus molecular subtypes (CMSs) [33]. KRAS mutation status has been used as a "molecular" predictor of efficacy for targeted therapy with epidermal growth factor receptor monoclonal antibody and has become class I evidence for clinical treatment. Patients with wild-type and G13D-mutant phenotypes can benefit from this type of drug therapy [9]. A new generation of KRAS mutation inhibitors has been used in the clinic; the first KRAS inhibitor sotorasib (AMG510) [34] became available in 2019, and the FDA approved adagrasib (MRTX849) for patients with the KRAS G12C mutation in 2021 [35]. We found that the expression level of OSBPL3 was significantly elevated in samples with KRAS mutations. Elevated OSBPL3 expression is unlikely to induce KRAS mutations. So, is this due to activated RAS signaling that enhances OSBPL3 gene expression? Recent studies have identified gene expression changes triggered by KRAS mutations, and some studies have shown that KRAS mutations increase the expression of VEGFR1 and VEGFR2 in CRC and lung adenocarcinoma, but the mechanism of this association remains unclear [36, 37]. Or is it because some complementary signaling pathways play an important role in tumorigenesis progression [38], which remains to be elucidated by future studies. The high level of OSBPL3 expression indicates that it may be a primary screening indicator for KRAS-mutated patients receiving KRAS inhibitors, and if the sample size is large enough, the clinicopathological characteristics of patients with KRAS mutations and high OSBPL3 expression can be analyzed to better characterize them. Mutations in the NRAS gene were not detected in this study. This may be because the proportion of NRAS mutations in CRC is approximately 2%–6% [39], which is much lower than that of KRAS. Additionally, there are limitations in the detection methods, as next-generation sequencing (NGS) methods detected RAS mutations in approximately 13% more patients [40]. The RAS pathway signature is superior to KRAS mutation status in predicting the dependence on RAS signaling [41]. Therefore, mutations in other members of the RAS pathway, not just the RAS gene, may play the same role in signaling. OSBPL3 may also be regulated by non-RAS pathways; for example, lncRNA MIR4435-2HG may regulate OSBPL3 expression via pathways such as the P38/MAPK pathway and the VEGF pathway [42]. Therefore, it is speculated that OSBPL3 does not facilitate colorectal carcinogenesis exclusively through the RAS pathway. Furthermore, although there is a correlation between KRAS mutations and OSBPL3, it is unclear whether OSBPL3 affects tumor biology regardless of KRAS status. This also deserves further study.

## Conclusions

In summary, our preliminary study demonstrated that OSBPL3 is upregulated in CRC and negatively correlates with the degree of differentiation. In addition, it may affect cell progression in CRC through the activation of RAS. Moreover, we found a significant correlation between OSBPL3 overexpression and high Ki-67 expression. Therefore, OSBPL3 may serve as a molecular marker for CRC diagnosis and progression and may be a new potential therapeutic target for CRC.

## Supplementary Information

**Additional file 1**.** Table 1**. Metadata Template.

## Data Availability

The datasets used during the current study are available from the corresponding author on reasonable request. The data of OSBPL3 mRNA expression detected by qRT-PCR has been deposited on Github (https://github.com/1061144079/project). Besides, the data can be viewed in the 'Additional file 1' of the article. The following databases were used for the data analysis:.TIMER2.0 (http://timer.cistrome.org/), GEPIA2 (http://gepia2.cancer-pku.cn/#index), CPTAC (http://ualcan.path.uab.edu/analysis-prot.html), OncoLnc ((http://www.oncolnc.org/).

## Abbreviations

CRC: Colorectal cancer
OSBP: Oxysterol-binding protein
ARMS: Amplification refractory mutation system
OS: Overall survival
PFS: Progression-free survival
PCR: Polymerase chain reaction
